# Predicting Properties from Near-Infrared Spectra with Machine Learning for Improved Polyolefin Differentiation

**DOI:** 10.1021/acspolymersau.5c00131

**Published:** 2026-01-29

**Authors:** Shuaijun Li, Robert J. S. Ivancic, Bradley P. Sutliff, Derek Huang, Enrique Blázquez-Blázquez, Tyler B. Martin, Kalman B. Migler, Debra J. Audus, Sara V. Orski

**Affiliations:** 1 Materials Science and Engineering Division, 10833 National Institute of Standards and Technology, Gaithersburg, Maryland 20899, United States; 2 Department of Physics and Institute for Soft Matter Synthesis and Metrology, Georgetown University, Washington, D.C. 20057, United States; 3 Institute of Polymer Science and Technology, ICTP-CSIC, Juan de la Cierva, 3, Madrid 20866, Spain

**Keywords:** machine learning, near-infrared spectroscopy, property prediction, polyolefin sorting, plastic recycling, model interpretability

## Abstract

As the scale and variety of plastics produced continue to grow, plastics recycling will require innovative solutions. The industrial state-of-the-art sorting technology, near-infrared (NIR) spectroscopy, as currently used, cannot effectively differentiate polyolefins, the single largest class of polymers by volume. Chemical similarity combined with architectural diversity in polyolefins stymies subclass delineation, such as differentiating low-density polyethylene from high-density polyethylene, due to their spectral similarity and chemical overlap. To address this challenge, we use machine learning (ML) to directly predict density, crystallinity, and short-chain branching from NIR spectra, enabling property-based sorting for more effective recycling. After testing a variety of ML models, we find that partial least squares regression provides high prediction accuracy with model simplicity. Since the resulting model leverages the correlated intensities, we develop a method to enhance interpretability by identifying the most important wavenumbers for property prediction, which we then relate to known polyolefin CH_3_ NIR vibrational absorption bands. This approach provides a linkage between ML model predictions and the underlying polyolefin chemistry and confirms that our models effectively capture spectrum–structure–property relationships in polyolefins, reinforcing the fundamental role of polymer chain structure in determining properties. These findings significantly contribute to the understanding of polyolefin differentiation using NIR spectroscopy, which could inform future advancements in property-based sorting strategies for plastic recycling efficiency.

## Graphical abstract

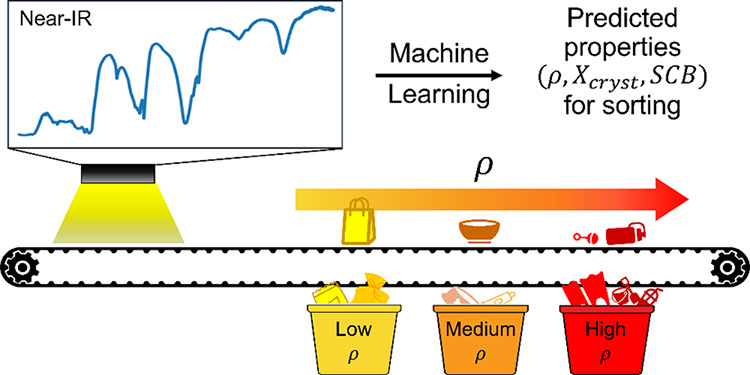

## 1 Introduction

Polyethylene (PE) and polypropylene (PP) represent 42% of the volume of all US plastics produced^1^ with wide use in automotive parts, furniture, housewares, and packaging, and specialty material applications, such as biomedical implants.^2^ The versatility of polyolefins arise from their wide range of material properties, driven by interdependent structural parameters such as monomer and comonomer content, branching content and type, molecular distributions (e.g., branching distributions, molecular mass distributions), and formulations (e.g., blends).^3^ Furthermore, polyolefin chain conformations are influenced by structural properties, material processing, and process history, which impact bulk properties such as density and crystallinity. Density is often used to grade polyolefins, and crystallinity contributes to the rigid, impact resistance (with high crystallinity) or the flexible character (more amorphous) of each semicrystalline polyolefin material. Several reviews in the literature have described the breadth of commercial polyolefin grades available.^4–7^

Despite their ubiquitous use, polyolefin recycling remains challenging.^8,9^ The American Society for Testing and Materials (ASTM) resin identification codes (RIC)^10^ only broadly categorize polyolefins, which leads to heterogeneous bales. For instance, Smith et al. analyzed 23 high-density polyethylene (HDPE) resins with RIC #2, revealing 56 characteristics that varied significantly across samples when examined through Fourier transform infrared (FTIR) spectroscopy, differential scanning calorimetry (DSC), thermogravimetric analysis, rheology, color analysis, and mechanical testing.^11^ This high level of heterogeneity clearly illustrates why current sorting practices struggle to produce consistent, high-quality recycled streams. Achieving maximum recycling and reprocessing rates requires significant improvements in sorting accuracy and efficiency to minimize feedstock heterogeneity and incentivize greater postconsumer resin incorporation. Therefore, there is a critical need to develop more precise methods for identifying and sorting polyolefins based on their material properties.

Machine learning (ML) has been increasingly applied to polymer property prediction and recycling, with recent reviews outlining key challenges and opportunities.^12,13^ Researchers have explored various spectroscopy methods combined with ML, such as mid-infrared (MIR) spectroscopy, Raman spectroscopy, and hyperspectral imaging, among others^14–17^ to predict polymer properties, to evaluate postconsumer recyclate viability, and to characterize biomass and waste material.^11,18,19^ For example, Tao presented a fast and effective method for characterizing biomass and waste using attenuated total reflectance (ATR) – FTIR spectroscopy and ML models,^19^ while Stavinski et al.^14^ and Zinchik et al.^20^ explored the application of MIR spectroscopy combined with ML to improve classification of postconsumer plastic waste. While these spectroscopic techniques are attractive because they can offer richer chemical information and potentially higher resolution for material identification, they pose significant challenges for industry applications due to low acquisition speeds, background fluorescence, or high cost.^15^

One potential method for improving sorting is to leverage NIR spectroscopy. NIR spectroscopy has emerged as the industrial state-of-the-art technique for medium to large volume plastic waste sorting due to its rapid, nondestructive, and efficient identification capabilities between different families of polymers such as polyethylene terephthalate, HDPE, and others categorized by the RIC.^21,22^ By more accurately identifying and separating these polymer types, recyclers can streamline processing, reduce contamination, and ultimately produce higher-quality recyclate suitable for reuse. However, distinguishing different members of the polyolefin family effectively using NIR alone remains challenging due to overlapping spectral features arising from their chemically similar structures.^23^ Researchers have explored various methods to improve NIR sorting, addressing these challenges and enhancing its accuracy for polyolefin separation. Sato et al. investigated NIR spectra of PE samples, including HDPE, low-density polyethylene (LDPE), and linear low-density polyethylene (LLDPE) to predict their physical properties such as density, crystallinity, and melting points.^24^ They used principal component analysis (PCA)^25^ for differentiating PE subclasses and partial least squares regression (PLSR)^26^ to build calibration models predicting the physical properties. They found that the first three principal components (PCs) accounted for more than 97% of the variance in NIR intensities and that the three types of PEs can be classified into three clusters considering the first and third PCs. They also achieved correlation coefficients for predicting these three properties and found that the spectral resolution is not critical in predicting accuracy. Bredacs et al. explored the use of various infrared (IR) spectroscopic techniques including ATR-FTIR, NIR, and dual-comb spectroscopy to predict density and melt flow rate of PE materials, with the goal of improving mechanical recycling of postconsumer PE products.^27^ Their results showed that NIR could broadly classify PE materials by density. However, these studies were limited to PE samples, relied on conventional chemometric methods and did not explore model interpretation, leaving key spectral contributions to property prediction largely unexplored. Furthermore, a critical scientific gap remains as it is not only the predictions that are required but also an understanding of which spectral regions drive those predictions. This is essential for insight and trust in model generalization.

In our previous work, we investigated the correlation between NIR spectra and bulk properties of polyolefins via dimensionality reduction techniques.^28^ First, functional PCA^29^ was applied to NIR spectra of polyolefins, including PP, and separation among various PE subclasses was successfully observed. Then, to further explore the relationship between the extracted spectral features and polymer properties, we identified strong correlations between NIR spectral data of PE with density, crystallinity, and short-chain branching (SCB). Our findings underscored the potential for NIR spectroscopy-based sorting of polyolefins by material properties. However, this study did not consider blends of polyolefins, nor did it make quantitative property predictions, which is essential for fine-grained sortation. More recently, we further expanded the use of ML based pipelines to optimize NIR data preprocessing and classification, achieving over 95% accuracy in differentiating polyolefins when considering existing class distinctions (HDPE, LDPE, LLDPE, PP).^30^ However, these advances primarily address class distinctions but do not capture the continuous nature of polymer properties that influence recyclability, highlighting the need for further development toward high-value, property-based sorting.

To improve NIR-based polyolefin differentiation for refined sorting, in this work, we employed ML techniques to predict the material properties of both nonblended and blended polyolefins, including density, crystallinity, and SCB from NIR spectra. These properties are critical for effective sorting of polyolefin waste as they directly influence the polymer’s mechanical and thermal behavior, which are essential for determining its suitability for various recycling processes and ultimate end use. As detailed in the **Methods** section, we have collected various types of polyolefins, including HDPE, LDPE, LLDPE, and PP. Additionally, we considered blends of HDPE and PP, as well as blends of HDPE and LDPE. After applying spectral preprocessing, we trained and tested a variety of supervised ML models and found PLSR provides the simplest model with low prediction error. To interpret the ML predictions, we developed a method that identifies a small number of important wavenumbers contributing to each predicted property and found that key spectral regions strongly correlated with known polyolefin absorption bands associated with polymer branching and chain-end structures. These spectra-chemistry linkages not only validated the robustness of our ML approach but also improved our understanding of how polymer molecular structures influence macroscopic properties. Therefore, our findings lay the foundation for more effective recycling practices via property-based sorting, a critical step toward achieving a circular plastics economy.

## 2 Methods

### 2.1 Materials

We collected a total of 39 samples from diverse sources[Fn fn1] including 10 commercial samples, 9 from the Hawaii Pacific University Polymer Kit 1.0, and 4 PE Standard Reference Materials from the National Institute of Standards and Technology (NIST): SRM 1473, 1474, 1475, and 1476. Among these samples, 7 were HDPEs, 8 were LDPEs, 2 were LLDPEs, 5 were PPs (including 2 PP-*co*-PE that contain mostly PP). All samples were provided in pellet form. Each sample type was classified according to the commercial material specification sheets. These samples were previously analyzed in our earlier work.^28^ To further expand the sample space, blends of HDPE and PP, as well as blends of HDPE and LDPE were prepared using a twin-screw extrusion method.^31,32^ These blends were created with incremental compositions of 0.1 mass fraction for both the HDPE/PP blends and the HDPE/LDPE blends. For the HDPE/LDPE blends, compositions corresponding to mass fractions of 0.4 and 0.6 were not included due to unavailability. Detailed information on the blending of HDPE/PP and HDPE/LDPE can be found in the Supporting Information (S1). The raw data^33^ and code^34^ for the measurements and analyses described herein are publicly available in order to ensure reproducibility.

### 2.2 Measurements

NIR measurements were conducted using a Nicolet iS50 NIR module from Thermo Fischer Scientific. The measurements were performed at a resolution of 4 cm^–1^ with an accumulation of 32 scans, utilizing an integrating sphere and an indium gallium arsenide detector. To enhance the robustness of subsequent ML models, six spectral replicates were collected for each sample, capturing potential variability in measurements. In total, 39 samples yielded 234 NIR spectra for model development. Density measurements were carried out using a Mettler Toledo density kit for the MS-104S balance, following ASTM Standard D792–20. The samples were weighed in air and isopropyl alcohol for the density calculation. Each measurement was repeated at least twice using different pellets, and the mean and standard deviation were reported for each sample. Crystallinity measurements were performed on a TA Instruments 2500 differential scanning calorimeter, with a heating rate of 10 °C/min in hermetic aluminum pans under a nitrogen flow of 50 mL/min. The crystallinity was evaluated dividing the enthalpy of melting Δ*H*_*m*_ from the first heating cycle by the heat of fusion Δ*H*_*m*_^0^. Δ*H*_*m*_^0^ was taken as 293.6 J/g for PE and 207.1 J/g for PP samples based on literature.^35^ SCB content was determined using high-temperature size exclusion chromatography with a Polymer Char GPC-IR instrument equipped with an IR4 detector and calibrated against poly­(ethylene-*co*-1-octene) standards or known mixtures of PE/PP obtained commercially. In general, HDPE is effectively linear with negligible SCB; LLDPE contains SCB arising from α-olefin comonomers, and LDPE contains predominantly ethyl and butyl SCB arising from intramolecular chain transfer. More detailed information about experimental procedures for density, crystallinity, and SCB measurements can be found in our previous work.^28^ Molecular mass measurements for the nonblended samples have been reported previously and are available in the NIST data repository (Data/AdditionalMeasurementData.csv).^36^ The molecular mass of the HDPE and PP used to prepare the HDPE/PP blends are reported in ref [^31^], and the corresponding molecular mass data for the HDPE and LDPE used to prepare HDPE/LDPE blends are provided in Table S2 in the Supporting Information (S2).

### 2.3 NIR Preprocessing

NIR spectra were preprocessed using the robust normal variate (RNV) method to minimize variations caused by sample shape and light scattering.^37–39^ Specifically, the interquartile range method was applied, using data points between the 25th and 75th percentiles to determine the effective mean and standard deviation for baseline correction and scaling. This approach enhances spectral consistency, improves the signal-to-noise ratio, and reduces the influence of outliers. Other common preprocessing methods were not considered due to their limitations. For example, multiplicative scattering correction requires a reference spectrum,^40^ which, if used in practice, would require multiple measurements for recyclers, slowing their process down. Standard normal variate is similar to RNV but is more sensitive to outliers and, thus, is a less robust choice for ML.^37^

After applying RNV preprocessing, spectra are further normalized so that their values at each wavenumber have zero mean and unit standard deviation, a common preprocessing step for ML. This standardization ensures consistency across samples, making the data more comparable and improving the performance of predictive models. Figure 1a presents example NIR spectra for different types of polyolefins. Figure 1b shows the RNV corrected spectra, which reduces the influence of sample shape and light scattering, bringing the spectra onto a more consistent scale while preserving key spectral features. The normalized spectra can be found in the Supporting Information (S3). Spectra were visually inspected for spikes, baseline distortions, and instrument artifacts, and no spectra were identified as outliers; therefore, all six replicates per sample were retained for analysis.

**Figure 1:**
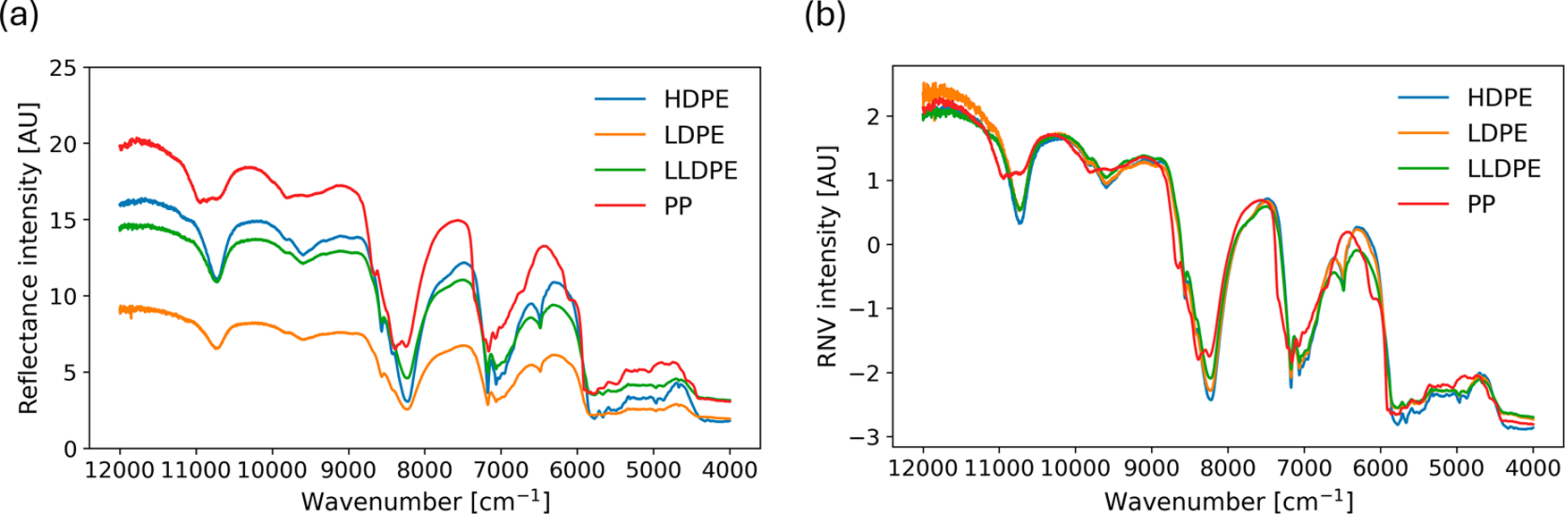
(a) Example NIR spectra; (b) RNV corrected spectra.

### 2.4 Machine Learning

We evaluated seven different supervised ML models for the prediction of density, crystallinity and SCB, including PLSR, principal component regression (PCR),^41^ least absolute shrinkage and selection operator (LASSO),^42^ linear regression (LR), random forest (RF),^43^ support vector regression (SVR),^44^ and Gaussian process regression (GPR).^45^ These models were chosen to represent a broad range of learning paradigms, including dimensionality reduction, linear, tree-based, and kernel-based regression methods, providing a comprehensive assessment of modeling strategies. All models were implemented using scikit-learn.^46^ More complicated methods such as neural networks were not considered due to the limited data set size.

To ensure robust evaluation, we employed nested 5-fold-cross validation. The outer loop split the data into training and testing sets, and the inner loop further divided the training set into training and validation sets with the validation set used to determine the model hyperparameters such as the optimal number of PLS components for PLSR. This approach provides an unbiased estimate of model performance. Hyperparameter tuning was performed using grid search in scikit-learn, with the objective of minimizing the root mean squared error (RMSE) on validation set.^46^ After the optimal hyperparameters were identified, the models were retrained on the combined training and validation data before being tested. To mitigate data set bias, we used stratified nested cross validation to ensure different sample types (such as HDPE, LDPE, LLDPE and PP) were distributed proportionally across train, validation, and test sets to reduce error due to biased data sets. Importantly, the six spectra replicates associated with each polymer sample were always kept together as a single sample unit and were never split across folds, preventing data leakage between training, validation, and testing. A full list of the hyperparameters for each model type and additional details are provided in the Supporting Information (S4).

### 2.5 Machine Learning Interpretability

Directly applying interpretability analysis such as SHapley Additive exPlanations (SHAP)^51^ or Local Interpretable Model-agnostic Explanations (LIME)^48,50^ would only help identify the most important PLS components. Since multiple wavenumbers are highly correlated (Supporting Information S5), even interpreting the most important PC is challenging. Instead, we took a different approach. Specifically, we identified a surrogate linear model that uses only intensities at a handful of wavenumbers and reproduces the PLSR predictions. In principle, this can be accomplished directly by brute force evaluating all possible models with one, two, three, or more wavenumbers. However, with over 4000 wavenumbers in our NIR spectral data, the number of possible feature combinations grows rapidly. For instance, evaluating all three-feature combinations would require testing approximately 59.5 billion models, and four-feature combinations would exceed 6 trillion, making brute-force selection computationally impractical beyond two or three features.

Instead, we first used LASSO to identify a reduced candidate pool (30–75 wavenumbers) and then performed the brute-force subset enumeration within this reduced space. For each property, we increased this candidate pool size until the surrogate linear model converged, ensuring LASSO did not affect the final surrogate model. This confirmed that 30–75 candidate window is sufficiently large to capture multiple correlated wavenumber clusters associated with broad NIR overtone bands, and expanding the candidate pool beyond this range did not change the optimal wavenumbers. To determine the size of the final surrogate model, we tracked the improvement in the RMSE (ratio of larger model to smaller model) for each candidate combination and chose the smallest set of wavenumbers at which this metric leveled off. This approach results in three important wavenumbers. Finally, we performed SHAP analysis on the surrogate linear model. We also computed the SHAP contribution arising from differences between the surrogate linear model and the PLSR model to assess the performance of the surrogate linear model. This approach allows us to identify a very small number of wavenumbers that are easy to interpret. Crucially, we then examine the correlation of these important wavenumbers versus all wavenumbers to give a more holistic view that is not dependent on the exact value of the important wavenumbers, thus making it more robust to small model variations. We compared the results from two different folds for one property and found that although the important wavenumbers sometimes changed (e.g., 8608 cm^–1^ to 8611 cm^–1^), they were highly correlated with one another yielding correlation plots that did not affect our analysis.

## 3 Results and Discussion

To enhance sorting beyond RIC HDPE (2), LDPE (4) and PP (5), we focus on predicting key material properties: density, crystallinity, and SCB, which influence resultant polymer properties and therefore recyclability. There are two major challenges to this approach. First, these properties are time-consuming to measure for recyclers. Second, NIR spectra exhibit broad and overlapping peaks resulting from combination and overtone bands, leading to high correlations among NIR intensities at different wavenumbers.

### 3.1 Model Selection

To overcome these challenges, we utilized ML. Since the best ML model often cannot be determined a priori,^49^ we considered a variety of different ML models. Figure 2 presents the performance of these models for predicting density, crystallinity, and SCB. Here, we measured performance using the RMSE on test data, as the RMSE is sensitive to outliers and can be directly compared with measurement uncertainty (the blue dashed line). We do not expect model error to drop below the measurement uncertainty of our samples, nor should it, as that could be interpreted as an overfitting of reasonable measurement error.

**Figure 2:**
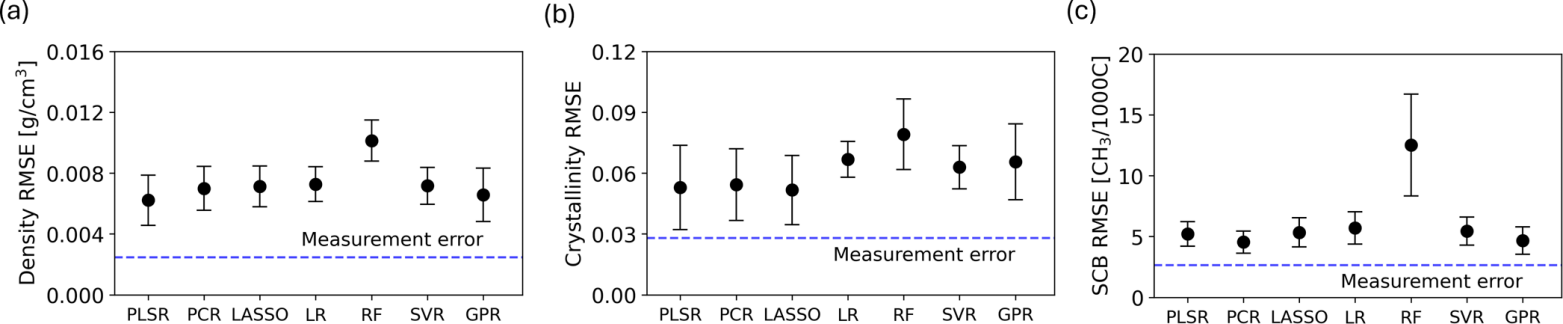
Averaged model performance for density, crystallinity, and SCB across the five test sets. Models include PLSR, PCR, LASSO, LR, RF, SVR, and GPR. Error bars represent one standard deviation of the RMSE from the five test sets arising from 5-fold cross validation. The blue dashed line represents measurement uncertainty.

All the models with the exception of RF achieved strong predictive capability across all three properties. Among these models, PLSR emerged as the most suitable due to its model compactness. Both PLSR and PCR incorporate dimensionality reduction to address the large number of correlated spectral features. PCR performs dimensionality reduction through PCA, which considers only NIR intensity values. PCA results demonstrated clear clustering of polyolefin classes and revealed a distinct trend from PP to PE (see Supporting Information S6), with the first three PCs capturing 95.1% of the variance. However, PLSR further enhances predictive capability by constructing latent variables that directly maximize covariance between spectral features and polymer properties. This optimized dimensionality reduction allows PLSR to achieve strong predictive accuracy with fewer components compared to PCR. Specifically, for predicting density, crystallinity, and SCB, PLSR required 9–15 components, whereas PCR needed 18–26 PCs. The variation of RMSE on validation sets as a function of the number of PLS components is provided in Supporting Information S7. LR used all features (wavenumbers) without regularization or dimensionality reduction, which resulted in overfitting (see Supporting Information S8). LASSO, which selects specific wavenumbers directly, generally required 12–121 parameters. While LASSO is inherently more interpretable, the large number of correlated intensities prevents direct analysis. The kernel methods, SVR and GPR, perform well but use the entire spectra. A detailed number of features used for ML prediction for the three properties is presented in Table S4 in the Supporting Information (S9).

The strong performance of linear models (PLSR, PCR, LASSO, and LR) is consistent with the spectroscopic principles underlying NIR reflectance measurements. The Beer–Lambert law describes a linear relationship between spectra absorbance and chemical composition. This linear relationship, generally, is preserved when the NIR reflectance spectra are converted to absorbance.^50^ As a result, linear models can effectively capture the dominant variance in spectral intensity associated with changes in SCB, which is related to chain methyl content.

### 3.2 Importance of Inclusion of Blends

Having identified PLSR as the preferred model, we also tested the importance of the inclusion of blends in our training data set. As detailed in **Materials**, we added two different types of blends (HDPE/PP and HDPE/LDPE) compared to prior work as we anticipated the presence of blends would be critical for accurate prediction. To test this hypothesis, we trained ML models only on nonblended polyolefins and then evaluated their performance on the blend samples, rather than training and testing on the full combined data set. As shown in Supporting Information S10, this exclusion of blends leads to a substantial increase in RMSE, indicating that incorporating blend samples into the training set is essential for achieving reliable predictive performance.

### 3.3 PLSR Property Prediction

Figure 3 presents parity plots illustrating the predictive performance of PLSR in estimating density, crystallinity, and SCB on training and testing data sets. Each plot represents one of the folds from a 5-fold nested cross-validation procedure. Each sample included six spectral replicates to enhance model robustness against noise. The close alignment of data points along the diagonal line indicates strong agreement between predicted and measured values, with R^2^ values around or above 0.9 for all three properties. Some deviation from the diagonal suggests minor prediction errors, partially due to spectral variability. A comparison with prior work by Sato et al. showed that, although their PLSR models for homogeneous PE samples achieved very low RMSE values, around 0.002 g/cm^3^ for density and 3% for crystallinity, our models obtained high accuracy relative to the experimental measurement uncertainty. It is also worth noting that Sato et al. averaged replicate spectra before calibration and used leave-one-out cross-validation, thereby reducing measurement variability and yielding lower apparent RMSE values.^24^ In contrast, our model retained all spectra replicates and maintained sample grouping during nested cross-validation, providing a more rigorous estimate of predictive performance when only one spectrum is acquired. To further assess potential outliers and influential observations, we examined studentized residual–leverage plot (Supporting Information S11 Figure S7), which appeared reasonable.

**Figure 3:**
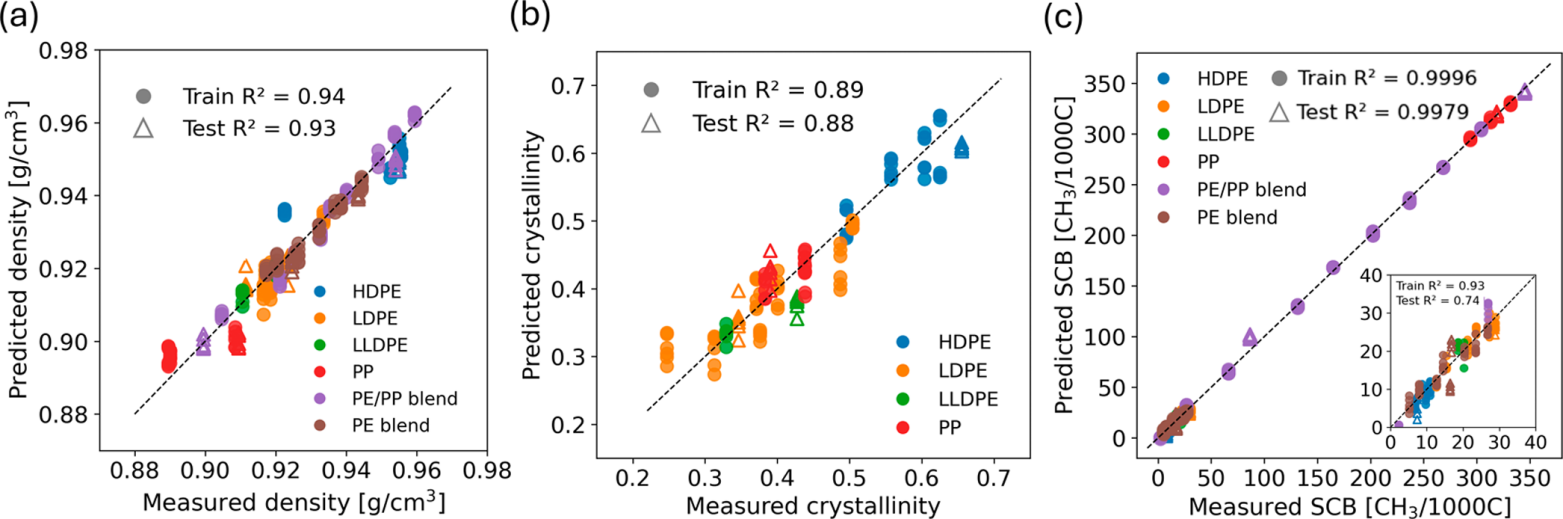
Parity plots show the goodness of fit of the PLSR model for train and test sets in predicting (a) density, (b) crystallinity, and (c) SCB. Test set RMSE values are 0.0052 g/cm^3^, 0.041, and 6.01 CH_3_/1000C for density, crystallinity and SCB, respectively. For crystallinity, only nonblend samples were used to avoid ambiguity. This is because PE and PP, as well as HDPE and LDPE, are immiscible, resulting in each polymer phase retaining its own crystalline structure rather than forming a single, well-mixed crystalline domain. As a result, a single, well-defined crystallinity value cannot be determined.

Analysis of individual polymer types revealed distinct clustering patterns across the property ranges. In Figure 3a, the density prediction showed excellent performance with HDPE samples predominantly occupying the higher density range, LDPE and LLDPE clustering in the mid-lower density regions, and PP samples at the lowest densities. As expected, PE/PP and HDPE/LDPE blends spanned almost the entire density range. It is important to note that these clusters do overlap due to variations within polymer grades. In Figure 3b, crystallinity predictions follow similar distribution patterns, with HDPE samples exhibiting the highest crystallinity values (0.55–0.65), while LDPE, LLDPE, and PP samples display lower crystallinity (0.30–0.50), consistent with their molecular structures. Figure 3c shows the exceptional accuracy of the model in predicting SCB. Here, HDPE clustered at the low SCB range, reflecting their linear structure. LDPE and LLDPE samples exhibited progressively higher branching levels, and PP appeared in the upper SCB range. The inset magnified the lower SCB region where minor deviations are observed. We note that the larger dispersion in the inset region between 0–30 SCB is expected. At low SCB levels, the HT-GPC/IR reference values have larger relative uncertainty, and the signal-to-noise ratio of the IR intensities is lower at lower concentrations in the chromatogram. Therefore, these effects increase the spread within this region.

### 3.4 Model Interpretation and Chemical Insight

Since many wavenumbers are highly correlated as demonstrated in the Supporting Information (S5), it is difficult to interpret PLSR predictions using its linear coefficients. To overcome this issue, we first developed a method to create a compact, surrogate linear model that mimics the PLSR model as discussed in **Methods**. Then, we applied SHAP to enhance model interpretability and explicitly understand the contribution of each selected wavenumber, as well as the remainder. This approach allows one to identify a small number of important wavenumbers for property prediction, while allowing for identification of correlated wavenumbers.

SHAP summary plots for SCB prediction is shown in Figure 4a. Each data point represents a sample at a specific wavenumber, where the *x*-axis denotes the SHAP value, reflecting the magnitude and direction of the feature’s contribution to the prediction. For a given sample, the sum of the SHAP values across all features is equivalent to the difference between the predicted value and the average predicted value across the entire data set. A positive SHAP value indicates that a feature increases the predicted value compared to the average, while a negative SHAP value indicates the feature decreases the predicted value. The magnitude of the SHAP value represents the strength of the feature’s impact. In the case of a linear model, SHAP values can be exactly computed via an analytical expression.^51^ The wavenumbers were ranked from most important to least important, and the contributions not captured by the surrogate linear model were relatively small as evidenced by the everything else category. Additionally, the color, ranging from blue to red, represents the magnitude of the NIR intensity at the corresponding wavenumber, revealing how spectral intensity values influenced the model’s predictions.

**Figure 4:**
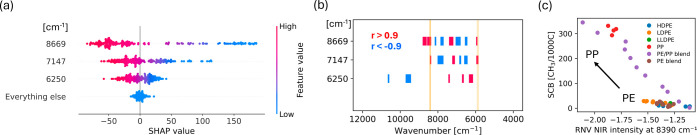
SHAP; correlation analysis; and NIR–property relationship for SCB. Note: Figures 4–6 share the same format. Each includes (a) a SHAP summary plot, (b) a correlation map with red indicating correlation >0.9 and blue < −0.9, and (c) a scatter plot comparing measured properties with NIR intensities. Orange vertical lines mark CH_3_ stretching NIR absorption bands for polyolefins taken from the literature, including the region 5848 cm^–1^ to 5917 cm^–1^ (C–H stretching, first overtone, CH_3_);^52^ and the bands at 8390 cm^–1^ and 8427 cm^–1^ (C–H stretching, second overtone CH_3_).^53^ These NIR overtone positions originate from the fundamental CH_3_ symmetric (2960 cm^–1^) and asymmetric (2870 cm^–1^) stretching vibrations for aliphatic hydrocarbons.^54^

It is important to note that while the identification of important wavenumbers provided critical insights into the spectral features contributing to property prediction, relying solely on these wavenumbers may overlook the broader spectral interactions that influence the model accuracy. Spectral data often exhibit interdependencies, where highly correlated wavenumbers can carry complementary or redundant information about the same molecular features. To capture these interactions, we analyzed the Pearson’s correlation coefficient between the intensities at the important wavenumbers and intensities at other wavenumbers across our entire data set. Figure 4b illustrates that wavenumbers significantly contributing to SCB predictions were strongly correlated with other wavenumbers, highlighted by the red and blue strips.

To further understand the implications of these correlations, it is necessary to relate the identified wavenumber regions to their underlying chemical functionalities. SCB content should be directly related to CH_3_ content because short-chain branches terminate with methyl groups, and more branching results in increased CH_3_ concentration. Indeed, we found that the first overtone CH_3_ stretching regions, marked by the orange rectangular region (5848 cm^–1^ to 5947 cm^–1^) and the second overtone CH_3_ stretching regions marked by vertical lines (8390 cm^–1^ and 8427 cm^–1^)^53,54^ were highly correlated with the important wavenumbers. For example, the 5848 cm^–1^ to 5947 cm^–1^ band, associated with terminal CH_3_ groups, overlapped with key spectral regions that are highly positively correlated with the important wavenumbers 8669 cm^–1^ and 7147 cm^–1^. Similarly, the CH_3_ stretching peaks at 8390 cm^–1^ and 8427 cm^–1^ also overlapped with other key spectral regions that were highly positively correlated with important wavenumbers 8669 cm^–1^ and 7147 cm^–1^, highlighting the influence of these bands on polyolefin property predictions. The presence of these reference lines in relation to highly correlated regions confirmed that our model captured the fundamental molecular features.

The direct relationship between spectral signal and SCB was further demonstrated in Figure 4c, which plots RNV processed NIR intensity values at 8390 cm^–1^ against the measured SCB values. A clear negative correlation was observed between NIR intensity and SCB content, with PP samples showing low NIR intensity corresponding to high SCB whereas PE samples showing high NIR intensity corresponding to low SCB values. This trend aligned well with the SHAP analysis results presented in Figure 4a, where important wavenumbers (8669 cm^–1^ and 7147 cm^–1^) had negative SHAP contributions to SCB prediction as their NIR intensity increases. Additionally, Figure 4b highlights a strong positive correlation between the intensity at 8390 cm^–1^ and these important wavenumbers. Considering reflectance NIR spectra, where absorption peaks face downward, higher SCB concentrations naturally correspond to lower NIR intensity values. Therefore, the observed trend in Figure 4c validated the SHAP findings, confirming that lower NIR intensities positively influence SCB predictions. However, Figure 4c also shows two distinct trends corresponding to PP and PE samples, underscoring that polymer type or blend composition cannot be reliably identified from intensity at a single wavenumber alone. This split behavior explains why single-band thresholding used in commercial NIR sorting can fail to reliably resolve composition differences. This limitation highlights the challenges faced by material recovery facilities that rely heavily on single point intensity measurements, thereby emphasizing the importance and practical value of our approach.

Figure 5 presents interpretability analysis of crystallinity prediction. The SHAP summary plot (Figure 5a) identified the most important wavenumbers contributing to crystallinity predictions. Since crystallinity in PE and PP arises from different structural mechanisms,^55,56^ two separate correlation maps were computed. The correlation map for PE is shown in Figure 5b and the correlation map for PP can be found in Supporting Information (S12). The PE correlation analysis (Figure 5b) highlighted spectral regions strongly correlated with the ML identified important wavenumbers. From Figure 5b, the most important wavenumber 7201 cm^–1^ was positively correlated with 8390 cm^–1^ CH_3_ stretching band. SHAP analysis in Figure 5a showed that increasing NIR intensity increases crystallinity at 8348 cm^–1^. This relationship was further highlighted in Figure 5c where the measured crystallinity is plotted against the RNV processed NIR intensity at 8390 cm^–1^. As previously mentioned, HDPE samples exhibit highest crystallinity, consistent with their linear molecular structure that facilitates efficient chain packing and crystallization, accompanied by higher NIR intensity values. In contrast, PP samples display intermediate crystallinity values, reflecting their regular and inherently different chain configuration that moderately restricts crystalline packing. LDPE and LLDPE display intermediate to lowest crystallinity values that reflect their crystalline nature resulting from branching that disrupts crystal formation.

**Figure 5:**
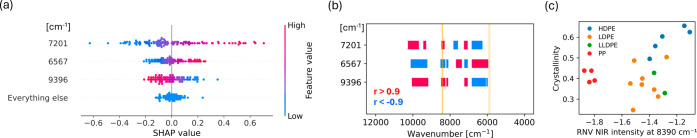
SHAP; correlation analysis; and NIR–property relationship for crystallinity.

The interpretability analysis for density is presented in Figure 6. Important wavenumbers were identified in Figure 6a. The intensity of the most important wavenumber, 8611 cm^–1^, is found to be positively correlated with density. This result can be understood in the context of the underlying chemistry. In Figure 6b, the correlation map revealed that the intensity at the important wavenumber of 8611 cm^–1^ had a strong positive correlation (r = 0.80) with the CH_3_ stretching band at 8390 cm^–1^, and in Figure 6c the CH_3_ stretching band at 8390 cm^–1^ is also found to be positively correlated with density. PE, particularly HDPE samples, exhibited higher density values with increasing NIR intensity, consistent with their linear, less branched molecular structures allowing closer chain packing, resulting in increased density. Conversely, PP samples displayed lower density values with decreased NIR intensities, reflecting their higher levels of branching and irregularity, preventing tight polymer chain packing and reducing density. Thus, again the consistency between SHAP analysis, spectral correlations, and experimentally measured density validated our ML approach.

**Figure 6:**
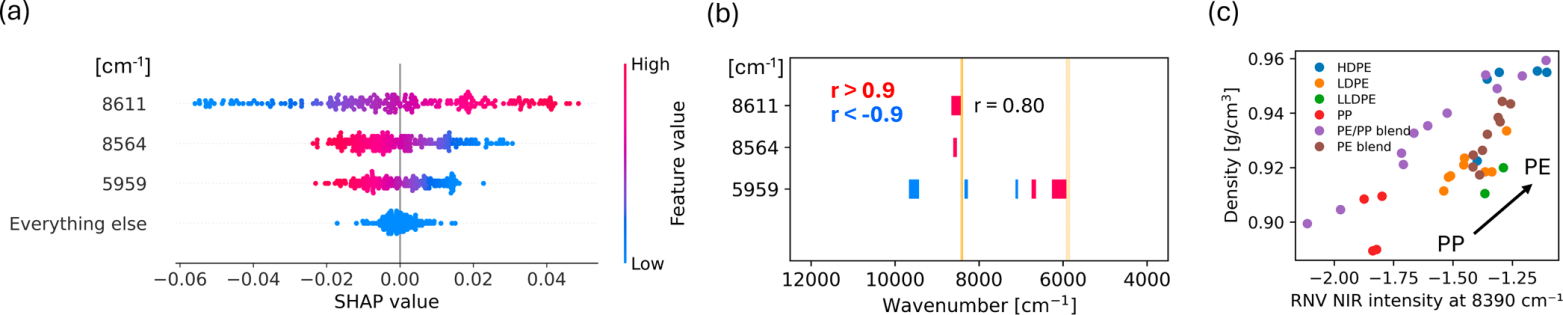
SHAP; correlation analysis; and NIR–property relationship for density.

Our analysis demonstrated how integrating ML with NIR spectroscopy enabled effective prediction and interpretation of polymer properties ranging from fundamental molecular features (SCB) through polymer structural organization (crystallinity) to macroscopic physical properties (density). Across these three properties, our findings revealed interconnected relationships aligned closely with fundamental polymer chemistry. Specifically, SCB served as the primary molecular feature that directly influenced crystallinity, which in turn affects density. The strong alignment between ML identified key spectral regions and known NIR absorption bands validated the ability of our ML models to extract meaningful chemical information from spectral data, emphasizing the fundamental role of CH_3_ vibrations in defining polymer properties.

Notably, variations in polymer chain architecture, such as branching density and methyl group placement, manifested in distinct yet interconnected spectral signatures. For instance, both PP and LLDPE contain methyl groups, but their structural arrangements and concentrations differ significantly: PP possesses alternating pendant methyl groups along its backbone arising from the propylene monomer, while LLDPE contains fewer terminal methyl groups at branch ends that are irregular spaced, and dependent on the comonomer (e.g., the type of α-olefin copolymerized with ethylene). These structural differences produced noticeable shifts in NIR intensities at CH_3_ bands, with PP exhibiting lower intensity at 8390 cm^–1^, indicating its higher methyl group density per chain length (Figure 4c). Similarly, branching density differences between HDPE and LDPE were clearly reflected as variations in the intensity of CH_3_ vibration bands. Thus, NIR spectra captured these subtle structural differences among closely related polyolefins, and our ML models were able to effectively leverage this sensitive polymer differentiation for accurate property prediction.

The identification and interpretation of these spectral variations underscore the robustness of our ML approach. By explicitly linking molecular structures to spectral features and physical properties, we demonstrated how ML methods could extract meaningful chemical insights from NIR spectral data. This comprehensive spectra-to-chemistry mapping not only enhances model interpretability but also significantly improves our understanding of how subtle variations in polymer molecular structure influence macroscopic polymer properties, ultimately supporting more informed and reliable polymer characterization.

### 3.5 Implications for Polyolefin Sorting and Recycling

This study demonstrated the capability of NIR spectroscopy combined with ML to predict key properties of polyolefins, including density, crystallinity, and SCB with high accuracy. Unlike traditional classification-based sorting approaches, we developed a property-based sorting strategy, which did not rely on broadly defined classes. By leveraging spectral data to predict material properties, this approach can be used for fine-grained sorting, ultimately leading to improved material compatibility in recycled streams and advancing plastic recycling.

While our study demonstrated the effectiveness of integrating ML with NIR spectroscopy for predicting polyolefins’ material properties, there are several critical hurdles prior to implementation at recycling facilities. For example, real-world scenarios introduce complexities such as contaminants, additives, and degradation, all influencing spectral signatures and challenging prediction accuracy. Additionally, in industrial conveyor-based sorting systems, plastics are moving on a belt, and the resulting motion-induced scattering variations may further degrade model accuracy relative to static benchtop spectra, requiring motion-robust calibration strategies. We also acknowledge that the present work was conducted on standard and commercial polyolefins and therefore reflects a controlled laboratory benchmark rather than a field validation. In practice, extending prediction accuracy to postconsumer recycled materials will require additional steps such as incorporation of degradation chemistries, expanded training sets including those with bimodal molecular mass distributions, and development of new models. We are currently acquiring spectra from postconsumer recycled samples as part of ongoing work toward evaluating model transfer to real waste streams, and this will be the focus of follow-up studies. Finally, although the present study focused on density, crystallinity, and SCB, other properties such as rheology are also important for value retention in recycled resins and are a logical future target for predictive modeling. Beyond this, integrating multimodal sensing, e.g., optical measurements, and transfer learning strategies offers an intriguing pathway for extending model generalization beyond laboratory conditions toward industrial field deployment.

## 4 Conclusions

Herein, we established a framework for integrating machine learning (ML) with near-infrared (NIR) spectroscopy to accurately predict key material properties of polyolefins, including density, crystallinity, and short-chain branching (SCB). Among various supervised ML approaches including dimensionality reduction, linear, tree-based, and kernel-based models, we found that partial least squares regression (PLSR) provided an advantageous balance between accuracy and simplicity.

To interpret the PLSR predictions, we developed a method to identify important wavenumbers at which the NIR intensities contribute the most to the PLSR prediction. This method makes use of the well-established SHAP analysis. We then found that these important wavenumbers were strongly correlated with molecular signatures such as CH_3_ vibrational absorption bands confirming the chemical significance of the important wavenumbers. This spectra-to-chemistry mapping demonstrated our model’s effective capability in capturing fundamental polymer structure–property relationships, reinforcing the fundamental role of polymer chain structure in determining material properties.

By applying ML to the industry-standard plastic waste sorting technique NIR spectroscopy, our ML approach enables rapid, nondestructive differentiation of polyolefins based on their physical properties rather than by polymer class (e.g., HDPE, LDPE). This is critical as NIR is already widely deployed in sorting facilities, our method has the potential to enhance material recovery using existing infrastructure, lowering barriers to adoption and accelerating impact. This combined ML NIR methodology therefore holds strong potential for improving plastic recycling accuracy and efficiency by enabling property-based polymer sorting, significantly advancing plastic recycling technologies. To further achieve these goals, our next step is to extend the current work to postconsumer resins. Ultimately, this work establishes a robust foundation for scalable polymer differentiation, enhancing recycling processes and advancing a sustainable circular economy.

## Supplementary Material



## Footnotes

* Any identification of commercial software, equipment, instruments, or materials in this work is for completeness and scientific understanding. Such identification does not imply recommendation or endorsement by NIST, nor does it imply that the materials or equipment identified are necessarily the best available for the purpose.

## Notes

Sample blending protocols; molecular mass of blends; normalized spectra example; machine learning hyperparameter; spectral correlation map; PCA score scatter plots; variation of RMSE on validation sets; model train and test RMSE comparison; number of features for machine learning models; blend generalization results; studentized–leverage residual plots; spectral correlation map for PP (PDF)
