# Supplementary material for: Predicting Properties from Near-Infrared Spectra with Machine Learning for Improved Polyolefin Differentiation
Source: ACS Polym Au. 2026 Jan 29;6(1):246–55. doi: 10.1021/acspolymersau.5c00131 (PMC12903461; doi:10.1021/acspolymersau.5c00131)
Supplement: Supplementary file 1 [file lg5c00131_si_001.pdf]

Supporting Information for Predicting Properties from Near-Infrared Spectra with Machine Learning for Improved Polyolefin Differentiation

Shuaijun Li[1,2], Robert J.S. Ivancic[1], Bradley P. Sutliff[1], Derek Huang[1], Enrique Blázquez-Blázquez[3], Tyler B. Martin[1], Kalman B. Migler[1], Debra J. Audus[1]*, Sara V. Orski[1]*

1. Materials Science and Engineering Division, National Institute of Standards and Technology, Gaithersburg, Maryland 20899
2. Department of Physics and Institute for Soft Matter Synthesis and Metrology, Georgetown University, Washington, D.C. 20057
3. Institute of Polymer Science and Technology, ICTP-CSIC, Juan de la Cierva, 3, 20866 Madrid, Spain

S1. Blends Sample Mixing Procedure and Short-Chain Branching (SCB) Quantification

For high-density polyethylene (HDPE) and polypropylene (PP) blends, samples were prepared by mixing HDPE and PP pellets in a twin-screw compounder manufactured by Daca Instruments. The blending was carried at 210 °C and 31.4 rad/s (300 rpm) for 5 minutes, after which the material was extruded into a long fiber and cut into short segments. Blends were created across the full range of HDPE/PP compositions in 10 % increments.[1] The HDPE/PP blends used in this study are identical to those used in ref [1].

For HDPE/LDPE blends, the components were mixed in a Haake Minilab twin-screw extruder with a 7 cm^3 volumetric capacity and co-rotating conical screws. Blending was conducted at 170 °C and 10.47 rad/s (100 rpm) for 5 minutes. After homogenization, the blends were compression molded into films using a Collin press. The films were formed between hot plates at 160 °C under 2.5 MPa pressure for 5 minutes, followed by rapid cooling to ambient temperature between water-chilled steel plates.[2]

Quantitative composition of polymer blends was determined using high-temperature gel permeation chromatography (HT-GPC). SCB content was experimentally measured for each blend

composition. Table S1 summarizes the measured SCB values and corresponding HDPE composition percentages for both HDPE/PP and HDPE/LDPE blends.

Table S1. Measured average SCB content for HDPE/PP and HDPE/LDPE blends at varying HDPE compositions.

HDPE composition (%)	0	10	20	30	40	50	60	70	80	90	100
Average SCB (HDPE/PP)	345.2	303.9	268.2	236.5	202.0	164.7	131.4	86.6	66.2	26.9	2.3
Average SCB (HDPE/LDPE)	26.7	20.4	16.8	23.5	—	14.5	—	12.7	16.45	7.9	5.2

S2. Molecular Mass of HDPE and LDPE Used for Preparing Blends

Table S2. Mass-average molecular mass (M_w) and dispersity (Đ) of the HDPE and LDPE.

	M_w (MALS) [g/mol]	Đ
HDPE	231700	6.83
LDPE	254400	10.14

The HDPE and LDPE homopolymers used to produce HDPE/LDPE blends were characterized by multi-angle light scattering (MALS) to determine their mass-average molecular mass (M_w) and dispersity (Đ). The results are summarized in Table S2.

S3. Normalized NIR spectra

After robust normal variate (RNV) transformation preprocessing, we normalized the spectra to have zero mean and unit standard deviation at each wavenumber. The resulting normalized spectra for representative non-blended polymers are shown in Figure S1.

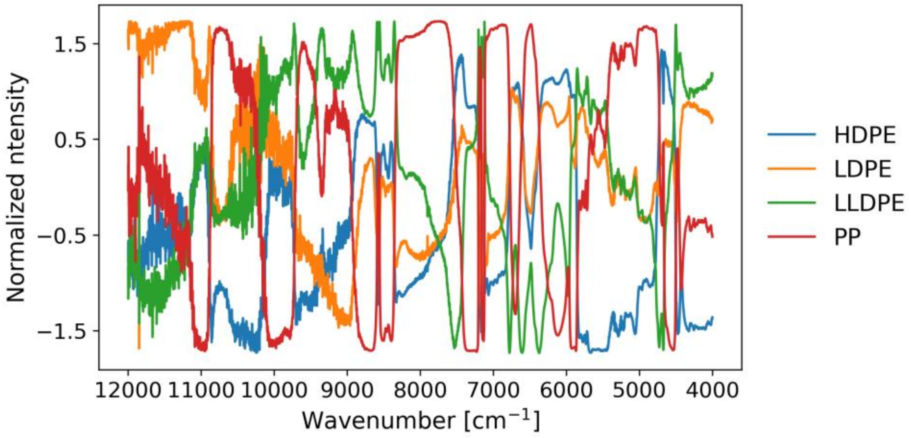

Figure S1. Normalized NIR spectra of HDPE, LDPE, LLDPE, and PP after RNV preprocessing.

S4. Machine Learning Hyperparameters

The hyperparameter ranges in Table S3 were selected based on commonly used values in the literature.[3,4] We also examined the best parameters derived from training data to ensure that they did not consistently lie at the boundaries of the specified ranges, which helped confirm that the search space was sufficiently broad. For models using latent variables including partial least squares regression (PLSR) and principal component regression (PCR), the number of latent variables was varied from 1 to 30 to capture the most informative variance. For least absolute shrinkage and selection operator (LASSO), regularization parameters were explored over logarithmic scales to efficiently cover several orders of magnitude. Random forest (RF) parameters were chosen to balance model complexity and performance. For support vector regression (SVR), we varied the regularization parameter C and the ε-insensitive margin ε to balance model complexity and noise tolerance. For Gaussian process regression (GPR), the prior and kernel parameters found via maximization of the log-marginal likelihood in scikit-learn following standard practice. Also, automatic relevance determination was not used due to the large number of features.

Table S3. Hyperparameters used in ML models for polyolefin property prediction.

Model	Hyperparameter	Values
PLSR	number of PLS components	1–30 (30 values)
PCR	number of PCs	1–30 (30 values)
LASSO	regularization parameter (α)	10^{-4}–10^{0} (10 values, log space)
LR	no hyperparameters (ordinary least squares regression)	N/A
RF	number of trees	[20, 50, 100, 200, 300]
	maximum tree depth	[2, 5, 10, 15, 20]
SVR	regularization parameter (C)	[0.1, 1, 10, 100]
	ε-insensitive loss width (ε)	[0.001, 0.01, 0.1, 1]

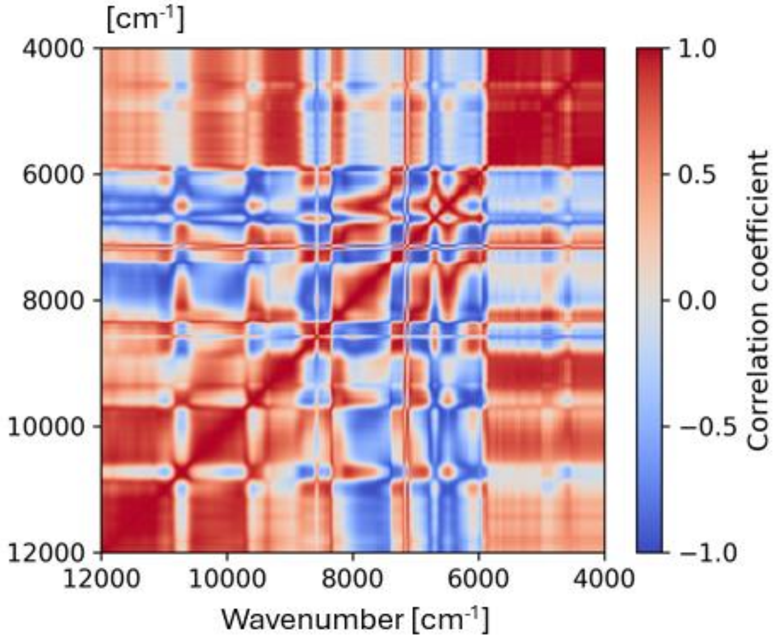

Figure S2. NIR intensity correlation coefficient heatmap across NIR wavenumbers.

S5. NIR intensities are highly correlated among different wavenumbers

To examine the correlations among different wavenumbers in the NIR spectral data, we constructed a correlation heatmap visualization. Prior to analysis, all NIR spectra were preprocessed using RNV

transformation to minimize physical effects. Pearson correlation coefficients were then calculated between all pairs of wavenumbers, resulting in a symmetric correlation matrix that quantifies the degree of linear correlation across the spectral range. In the heatmap visualization, color intensity reflects the strength of correlation. As shown in Figure S2, the NIR intensities exhibited strong correlations, both positive and negative, across the wavenumbers.

S6. PCA clusters similar classes and presents a trend from PP to PE

The first three PCs accounted for 95.1% of the total variance in the NIR data, effectively capturing the majority of the spectral information and demonstrating effectiveness of dimensionality reduction. The projection of PC scores onto the first two PCs (Figure S3(a)) revealed clear clustering patterns, highlighting meaningful distinctions among the polyolefin classes. PC score projection on the 2nd and 3rd PCs (Figure S3(b)) showed a clear compositional gradient trend from PP to PE. The clustering and clear compositional trends strongly indicated that the PC scores captured key chemical and structural variations inherent to these polyolefins. Therefore, the results illustrated that PCA effectively reduced the spectral data dimensionality while maintaining significant chemical interpretability. The identified clustering and compositional gradients provided meaningful insights into polymer classification and blend composition, directly contributing to the accurate prediction of physical properties through subsequent ML approaches.

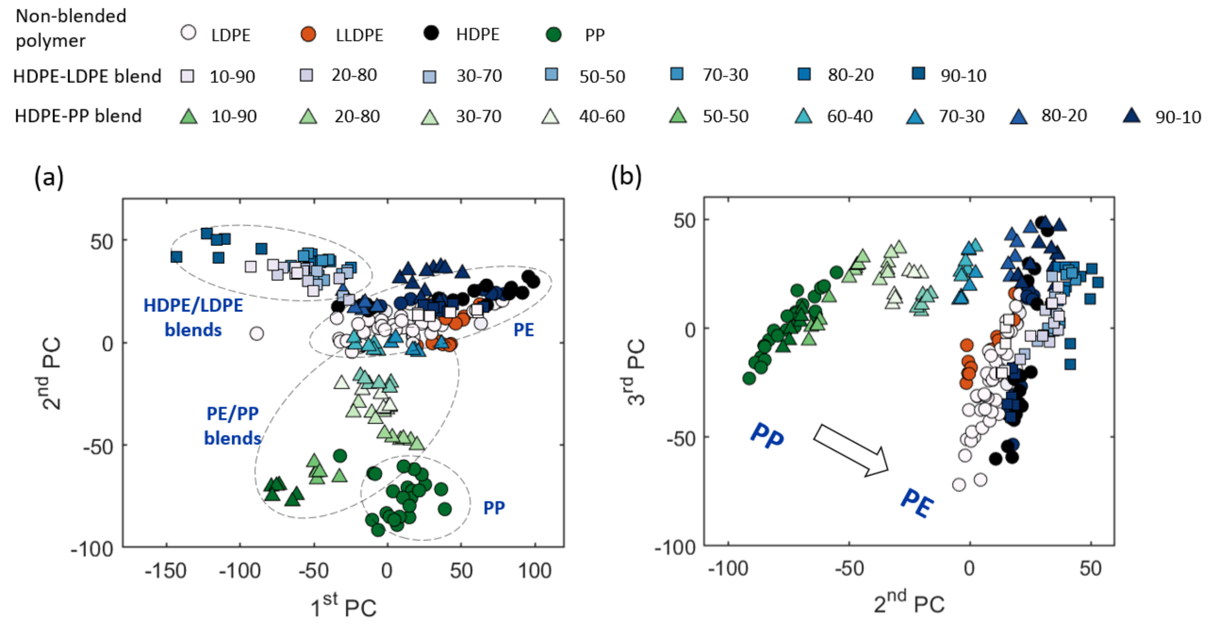

Figure S3. PC score scatter plots: (a) sample clustering on PC1 vs. PC2; (b) a clear trend from PP to PE on projection of PC2 vs. PC3.

S7. Variation of RMSE on Validation Sets with the Number of PLS components

To evaluate the appropriate model complexity for PLSR, we examined how the RMSE on validation sets varies as a function of the number of PLS components. For each property, 5-fold nested cross-validation was performed across a range of 1–30 PLS components. This yields 25 validation RMSE values for each PLS component, corresponding to all combinations of inner and outer folds. The mean RMSE and standard deviation across these 25 values were calculated and plotted to assess model accuracy and complexity. As shown in Figure S4, the RMSE decreases rapidly with the first few components, followed by a plateau region in which additional components yield diminishing improvements.

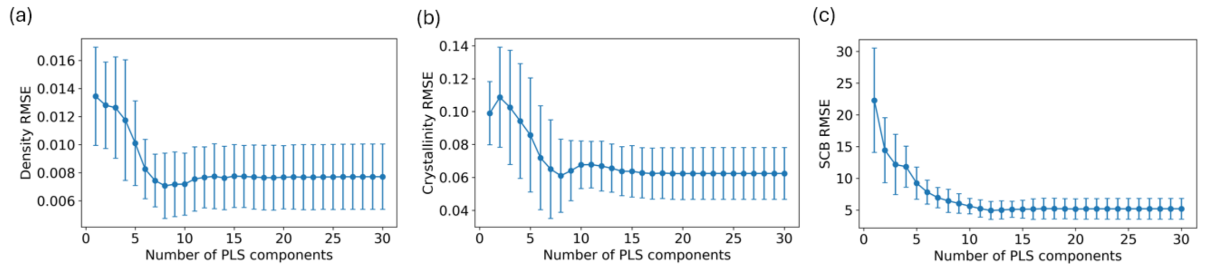

Figure S4. Variation of RMSE on validation sets with the number of PLS components for (a) density, (b) crystallinity, and (c) SCB predictions using PLSR.

S8. Comparing Root Mean Squared Error (RMSE) on Train and Test Data

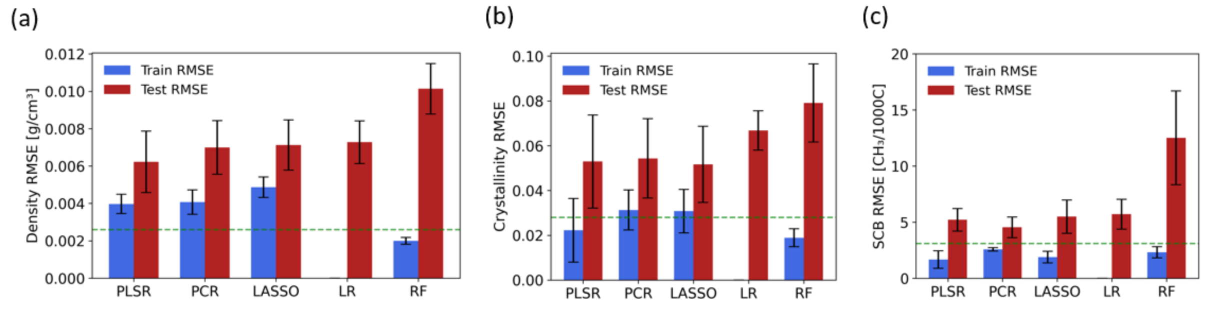

Figure S5. Comparison of RMSE for train and test datasets. The green dashed line indicates the measurement uncertainty.

Figure S5 illustrates the performance of PLSR, PCR, LASSO, LR, and RF models in terms of RMSE for both train and test datasets. It is worth noting that the nonlinear RF model exhibited higher RMSE values on test datasets as well as large gaps between train and test sets. The substantial gap between

train and test RMSE for RF model indicated overfitting and reduced generalization capability even after hyperparameter optimization. Notice also that the LR model almost had near-zero RMSE on the training sets but substantially higher RMSE on the test sets. This behavior reflects overfitting due to the use of all NIR wavenumbers without regularization, which compromises the model's ability to generalize to unseen data.

S9. Number of Features for ML Model

Table S4. Average number of features used in ML models for predicting SCB, crystallinity, and density.

	**PLSR**	PCR	LASSO	LR	RF	SVR	GPR
SCB	**15.2**	26.2	121.0	4149.0	1294.4	4149.0	4149.0
crystallinity	**11.2**	19.2	26.4	4149.0	573.6	4149.0	4149.0
density	**8.6**	17.8	11.8	4149.0	1862.0	4149.0	4149.0

S10. Train non-blended samples and test on blended samples

In the study evaluating the prediction of density and SCB in polyolefins, models were also trained on non-blended samples and tested on the blended samples. This approach allowed for evaluating the model's ability to generalize from a well-defined, uniform material (non-blended samples) to more complex compositions (blended samples). The results demonstrate notably higher RMSE values when models were

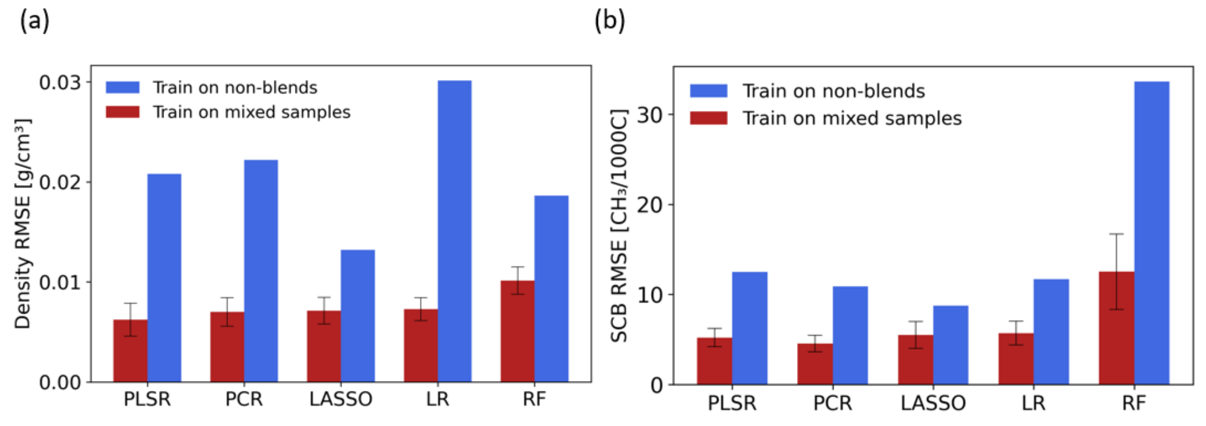

Figure S6. Comparison of test set RMSE for two modeling strategies: (1) training on non-blended samples (blue bars) and testing on blended samples; and (2) training on a combined dataset (red bars) comprising both non-blended and blended samples. (a) RMSE for density prediction; (b) RMSE for SCB prediction.

trained on non-blended samples and tested on blended samples, as shown in Figure S6, suggesting that including blend data in training significantly improved predictive accuracy.

S11 Studentized residual–leverage plot for SCB prediction

To identify potential outliers and influential observations in the PLSR model for density, crystallinity, and SCB prediction, we generated studentized residual–leverage plots. Leverage quantifies how unusual each spectrum is relative to the full dataset, with higher values indicating spectra that have greater influence on the model fit. Studentized residuals measure how well each spectrum is fitted by the model, with values outside ± 3 are usually considered outliers.

As shown in the figure, across all three properties, the majority of samples exhibit moderate leverage values and studentized residuals within ± 2. indicating that the models are not dominated by a small number of points. A few spectra lie outside the ± 3 range: one LLDPE spectrum for SCB and several HDPE spectra for density. These points have moderate leverage, indicating that they do not exert disproportionate influence on the regression coefficients. Overall, the plots in Figure S7 support that the PLSR models are reasonably well-behaved and are not driven by a few influential outliers.

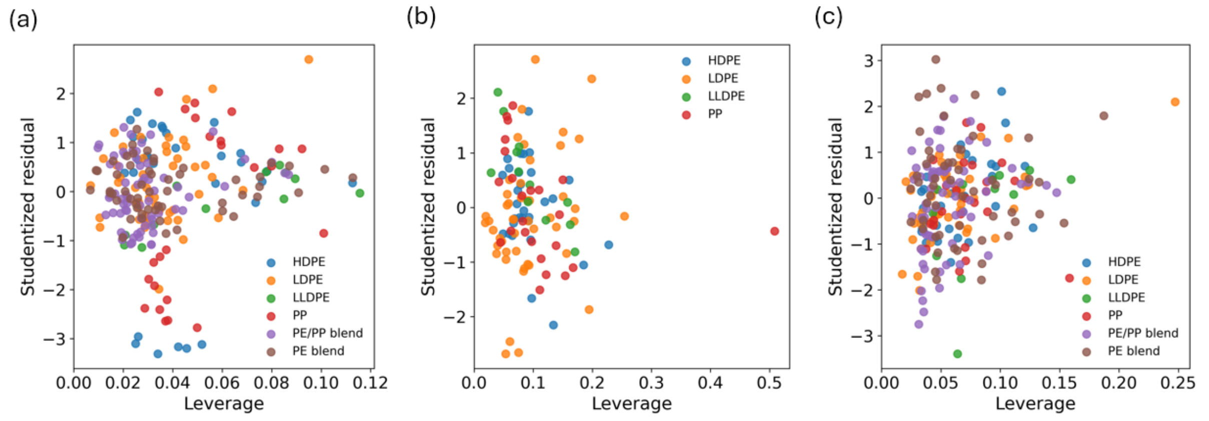

Figure S7. Studentized residual–leverage plot for (a) density, (b) crystallinity, and (c) SCB.

S12. Spectral correlation map for PP

Figure S8 presents the key spectral regions correlation map for PP crystallinity prediction. Similar to PE, important wavenumbers identified from ML were correlated across the full spectral range to highlight regions most strongly associated with PP crystallinity. The correlation patterns differ from those of PE, reflecting the distinct structural mechanisms underlying crystallinity in PE and PP, as discussed in the main text.

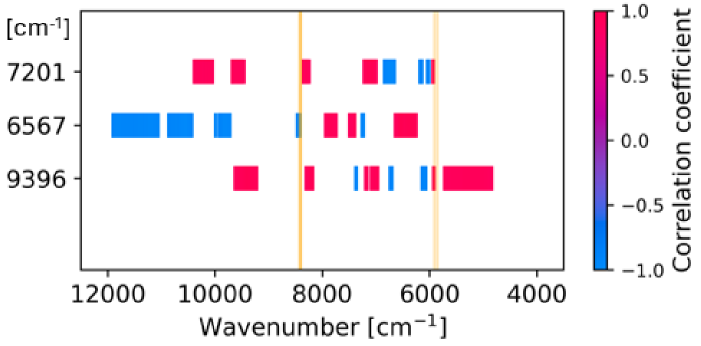

Figure S8. ML identified key spectral regions for PP.

References

1. Huang, D. E.; Kotula, A. P.; Snyder, C. R.; Migler, K. B. Crystallization kinetics in an immiscible polyolefin blend. *Macromolecules* **2022**, *55*, 10921–10932.

2. Blázquez-Blázquez, E.; Pérez, E.; Lorenzo, V.; Cerrada, M. L. Crystalline characteristics and their influence in the mechanical performance in poly (ε-caprolactone)/high density polyethylene blends. *Polymers* **2019**, *11*, 1874.

3. Probst, P.; Wright, M. N.; Boulesteix, A. Hyperparameters and tuning strategies for random forest. *Wiley Interdiscip. Rev.: Data Min. Knowl. Discov.* **2019**, *9*, e1301.

4. Tsirikoglou, P.; Abraham, S.; Contino, F.; Lacor, C.; Ghorbaniasl, G. A hyperparameters selection technique for support vector regression models. *Appl. Soft Comput.* **2017**, *61*, 139–148.
